# Effect of the Mediterranean diet on blood pressure in the PREDIMED trial: results from a randomized controlled trial

**DOI:** 10.1186/1741-7015-11-207

**Published:** 2013-09-19

**Authors:** Estefania Toledo, Frank B Hu, Ramon Estruch, Pilar Buil-Cosiales, Dolores Corella, Jordi Salas-Salvadó, M Isabel Covas, Fernando Arós, Enrique Gómez-Gracia, Miquel Fiol, Jose Lapetra, Luis Serra-Majem, Xavier Pinto, Rosa M Lamuela-Raventós, Guillermo Saez, Mònica Bulló, Valentina Ruiz-Gutiérrez, Emilio Ros, José V Sorli, Miguel Angel Martinez-Gonzalez

**Affiliations:** 1 Department of Preventive Medicine and Public Health, University of Navarra, Pamplona, Navarra, Spain; 2 Department of Nutrition, Harvard School of Public Health, Boston, MA, USA; 3 CIBER Fisiopatología de la Obesidad y Nutrición (CIBER OBN), Instituto de Salud Carlos III (ISCIII), Spanish Government, Madrid, Spain; 4 Harvard Medical School and Channing Lab, Brigham Women’s Hospital, Boston, MA, USA; 5 Department of Internal Medicine, Institut d’Investigacions Biomèdiques August Pi Sunyer (IDIBAPS), Hospital Clinic, University of Barcelona, Barcelona, Spain; 6 Department of Preventive Medicine and Public Health, University of Valencia, Valencia, Spain; 7 Human Nutrition Department, IISPV, Universitat Rovira i Virgili, Reus, Spain; 8 Cardiovascular and Nutrition Research Group, Institut de Recerca Hospital del Mar, Barcelona, Spain; 9 Department of Cardiology, University Hospital of Alava, Vitoria, Spain; 10 Department of Preventive Medicine, University of Malaga, Malaga, Spain; 11 Institute of Health Sciences IUNICS, University of the Balearic Islands, and Hospital Son Espases, Palma de Mallorca, Spain; 12 Department of Family Medicine, Primary Care Division of Sevilla, San Pablo Health Center, Sevilla, Spain; 13 Department of Clinical Sciences, University of Las Palmas de Gran Canaria, Las Palmas, Spain; 14 Lipids and Vascular Risk Unit, Internal Medicine, Hospital Universitario de Bellvitge, Hospitalet de Llobregat, Barcelona, Spain; 15 Department of Nutrition and Food Science, School of Pharmacy, XaRTA, INSA, University of Barcelona, Barcelona, Spain; 16 Department of Biochemistry and Molecular Biology, School of Medicine, University of Valencia, Valencia, Spain; 17 Department of Clinical Analyses, University Hospital of Valencia, Valencia, Spain; 18 Instituto de la Grasa, Consejo Superior de Investigaciones Cientificas, Sevilla, Spain; 19 Lipid Clinic, Department of Endocrinology and Nutrition, Institut d’Investigacions Biomèdiques August Pi Sunyer (IDIBAPS), Hospital Clinic, University of Barcelona, Barcelona, Spain; 20 Primary Care Division, Valencia Institute of Health, Valencia, Spain

**Keywords:** Mediterranean diet, Low-fat diet, Systolic blood pressure, Diastolic blood pressure, Controlled trial, PREDIMED trial, Monounsaturated fat, Dietary patterns, Olive oil, Nuts

## Abstract

**Background:**

Hypertension can be prevented by adopting healthy dietary patterns. Our aim was to assess the 4-year effect on blood pressure (BP) control of a randomized feeding trial promoting the traditional Mediterranean dietary pattern.

**Methods:**

The PREDIMED primary prevention trial is a randomized, single-blinded, controlled trial conducted in Spanish primary healthcare centers. We recruited 7,447 men (aged 55 to 80 years) and women (aged 60 to 80 years) who had high risk for cardiovascular disease. Participants were assigned to a control group or to one of two Mediterranean diets. The control group received education on following a low-fat diet, while the groups on Mediterranean diets received nutritional education and also free foods; either extra virgin olive oil, or nuts. Trained personnel measured participants’ BP at baseline and once yearly during a 4-year follow-up. We used generalized estimating equations to assess the differences between groups during the follow-up.

**Results:**

The percentage of participants with controlled BP increased in all three intervention groups (*P*-value for within-group changes: *P*<0.001). Participants allocated to either of the two Mediterranean diet groups had significantly lower diastolic BP than the participants in the control group (−1.53 mmHg (95% confidence interval (CI) −2.01 to −1.04) for the Mediterranean diet supplemented with extra virgin olive oil, and −0.65 mmHg (95% CI -1.15 to −0.15) mmHg for the Mediterranean diet supplemented with nuts). No between-group differences in changes of systolic BP were seen.

**Conclusions:**

Both the traditional Mediterranean diet and a low-fat diet exerted beneficial effects on BP and could be part of advice to patients for controlling BP. However, we found lower values of diastolic BP in the two groups promoting the Mediterranean diet with extra virgin olive oil or with nuts than in the control group.

**Trial registration:**

Current Controlled Trials ISRCTN35739639

## Background

In 2003, the Joint National Committee on Prevention, Detection, Evaluation, and Treatment of High Blood Pressure estimated that hypertension affects approximately 1 billion people worldwide [1]. This condition is a major risk factor for stroke, ischemic heart disease, and other chronic cardiovascular diseases (CVDs) [2]. In fact, the relationship between blood pressure (BP) and risk of CVD events is continuous, consistent, and independent of other risk factors [1]. Because of its high prevalence and its related conditions, hypertension is the leading individual risk factor for mortality, and is responsible for 7.6 million deaths per year [3]. Therefore, from a public heath perspective, approaches to tackle this condition are needed urgently.

Adopting a healthy lifestyle is a cornerstone of hypertension prevention and treatment, and a healthy diet represents a major lifestyle modification for BP control [1,4]. High-quality overall dietary patterns, such as the Dietary Approaches to Stop Hypertension (DASH) diet, can be of utmost importance in the prevention and treatment of hypertension [5]. Of these high-quality dietary patterns, one in particular has received much recent attention because of the growing evidence for its role in cardiovascular protection, namely, the traditional Mediterranean diet (MD) [6]. The MD is a traditional food pattern present in the olive oil-producing areas of the Mediterranean basin. Like the DASH diet, the traditional MD is rich in fruits and vegetables, but it also includes an abundance of legumes, a moderate intake of fish, dairy products, and wine, small portions of meat and poultry, and little consumption of candies (sweets) [7]. A key characteristic of this diet is the low amount of animal and *trans* fatty acids. Extra virgin olive oil (EVOO), the primary source of fat in the MD, along with plant foods and nuts, makes this diet ideal for health because these fresh foods undergo minimal processing, so they are rich in fiber, antioxidant polyphenols, and essential micronutrients and macronutrients. Recently, the PREDIMED primary prevention trial showed that a dietary intervention designed to foster adherence to the traditional MD significantly reduced the risk of CVD clinical end-points [8]. The reported reduction in CVD was most evident for stroke, a condition known to be highly dependent on BP. Therefore, one mechanism by which the traditional MD may exert its beneficial effect is in the control of BP. In fact, a recent meta-analysis reporting results from clinical studies supported a protective role for the MD on both systolic and diastolic BP. However, only two studies had a follow-up time beyond 2 years, and the largest of the two had a sample size of 605 subjects [9].

The aim of this study was to assess the long-term effect on BP of a dietary intervention to improve adherence to the traditional MD.

## Methods

### Design overview

The PREDIMED (Prevención con Dieta Mediterránea) study is a multicenter, randomized, parallel-group trial conducted in Spain (http://www.predimed.es). A detailed description of the methods and objectives of the PREDIMED trial can be found elsewhere [10]. Briefly, this trial was designed to assess the effects of the traditional MD on the primary prevention of CVD. The main outcome was an aggregate of non-fatal myocardial infarction, non-fatal stroke, or cardiovascular death. The trial was stopped because of early benefit by December 1, 2010 after a median follow-up time of 4.8 years [4].

The current work ascertains the long-term effect of the dietary interventions on changes in BP during 4 years of follow-up.

### Ethics approval

The protocol was written in accordance with the principles of the Declaration of Helsinki, was approved by the Institutional Review Boards at all study sites (for more detailed information, please check Additional file 1), and was registered at http://www.controlled-trials.com/ISRCTN35739639. Written informed consent was provided by all study participants.

### Setting and participants

Eligible participants were men (aged 55 to 80 years) and women (aged 60 to 80 years) who were free of CVD at study inception but at high cardiovascular risk because of the presence of either type 2 diabetes (T2D) or at least three major CVD risk factors, including current smoking, hypertension, high levels of low-density lipoprotein cholesterol, low levels of high-density lipoprotein cholesterol, overweight/obesity, or family history of premature coronary heart disease (CHD). Further details of the inclusion and exclusion criteria can be found in our previously published report [10]. Study candidates were selected from databases of primary care facilities. Of those who met entry requirements, 89% agreed to participate.

At baseline, participants completed a general medical questionnaire, a 137-item previously validated food-frequency questionnaire [11,12], the Minnesota Leisure-Time Physical Activity Questionnaire [13,14], and a 14-item screening questionnaire of adherence to the traditional MD [15].

### Randomization and interventions

During the period October 2003 to June 2009, 7,447 participants were enrolled in the study, and randomly allocated in a 1:1:1 ratio by means of a computer-generated random-number sequence to one of the three intervention groups: MD supplemented with EVOO (MD+EVOO), MD supplemented with mixed nuts (MD+nuts: walnuts, almonds, and hazelnuts), or the control diet (low-fat diet). The coordinating center constructed a computer-generated randomization table, with allocation concealment by opaque, sequentially numbered, sealed envelopes.

At baseline and quarterly thereafter, dieticians ran individual and group sessions, with no more than 20 participants, separately for each of the three groups. Group sessions were specific for each intervention group so that participants were assessed only for adherence to the diet to which they had been allocated. In the appropriate individual sessions, a 14-item dietary screening questionnaire was used to check for adherence to either of the MDs, and a 9-item dietary screening questionnaire was used to check for adherence to the control low-fat diet [15]. The questionnaire responses were used to personalize the intervention for each participant, and to negotiate dietary changes to upgrade adherence to either the MD or the low-fat diet. Participants in the two intervention groups were given supplementary foods at no cost: either EVOO (1 liter/week for the participant and their families) or mixed nuts (30 g/day: 15 g walnuts, 7.5 g hazelnuts, and 7.5 g almonds) according to their randomization group. Supplementation of these foods was intended to ensure high consumption of these key elements of the traditional MD, and to promote a better overall adherence to the target overall dietary pattern.

The control group received usual care and dietary counseling (including group sessions) aimed to increase their adherence to a lower-fat diet. The control group received non-food items as incentives throughout the study.

Energy restriction was not specifically advised nor was physical activity promoted in any of the three groups, and the interventions did not target sodium intake or sleep habits. Drugs were prescribed during regular medical care of the participants and were not influenced by the intervention.

### Outcomes and follow-up

At baseline and once yearly thereafter, trained personnel measured participants’ BP in each arm with a validated semiautomatic oscillometer (Omron HEM-705CP, Hoofddorp, the Netherlands) at three time points, separated by 2 minutes, while the participant was in a seated position after 5 minutes of rest. Arm circumference determined the cuff size, and BP was measured in the forearm at heart level. The average of the second and third measurement was recorded in the data collection form.

The mean of the systolic and diastolic BP measurements was also calculated [16,17]. The following values were considered extreme and were not taken into account for the analyses: systolic BP <70 mmHg or >260 mmHg, diastolic BP <40 mmHg or >135 mmHg, systolic BP changes >40 mmHg at 1 year, and diastolic BP changes >25 mmHg at 1 year.

For the present analysis, we have included information for 4 years of follow-up (median follow-up time 3.8 years) because the recruitment ended in 2009, and BP measurements were not available for a substantial number of participants beyond 4 years.

### Statistical analysis

Baseline characteristics are presented according to the intervention group, as mean (SD) for quantitative traits and n (%) for categorical variables.

All analyses were performed in accordance with an intention-to-treat approach. First, we assessed differences in changes in BP between groups during follow-up [18]. For participants with missing values of BP in the year 4 visit, we used the most recent available BP information. Second, we used generalized estimating equations to calculate mean differences in systolic and diastolic BP changes between the groups allocated to the MD+EVOO or MD+nuts versus the control group in crude analyses, and after adjustment for center, age, sex and baseline T2D, and, additionally for baseline number of anti-hypertensive drugs and baseline systolic or diastolic BP. We assumed an unstructured correlation matrix and calculated robust variance estimates. Third, we also used generalized estimating equations to calculate the percentage of participants with controlled BP levels (systolic BP <140 mmHg and diastolic BP <90 mmHg) during follow-up. Fourth, we used generalized estimating equations to ascertain the number of anti-hypertensive drugs that were prescribed during follow-up. Analyses were performed using STATA software (version 11.0; StataCorp, College Station, TX, USA).

## Results

**Figure 1:**
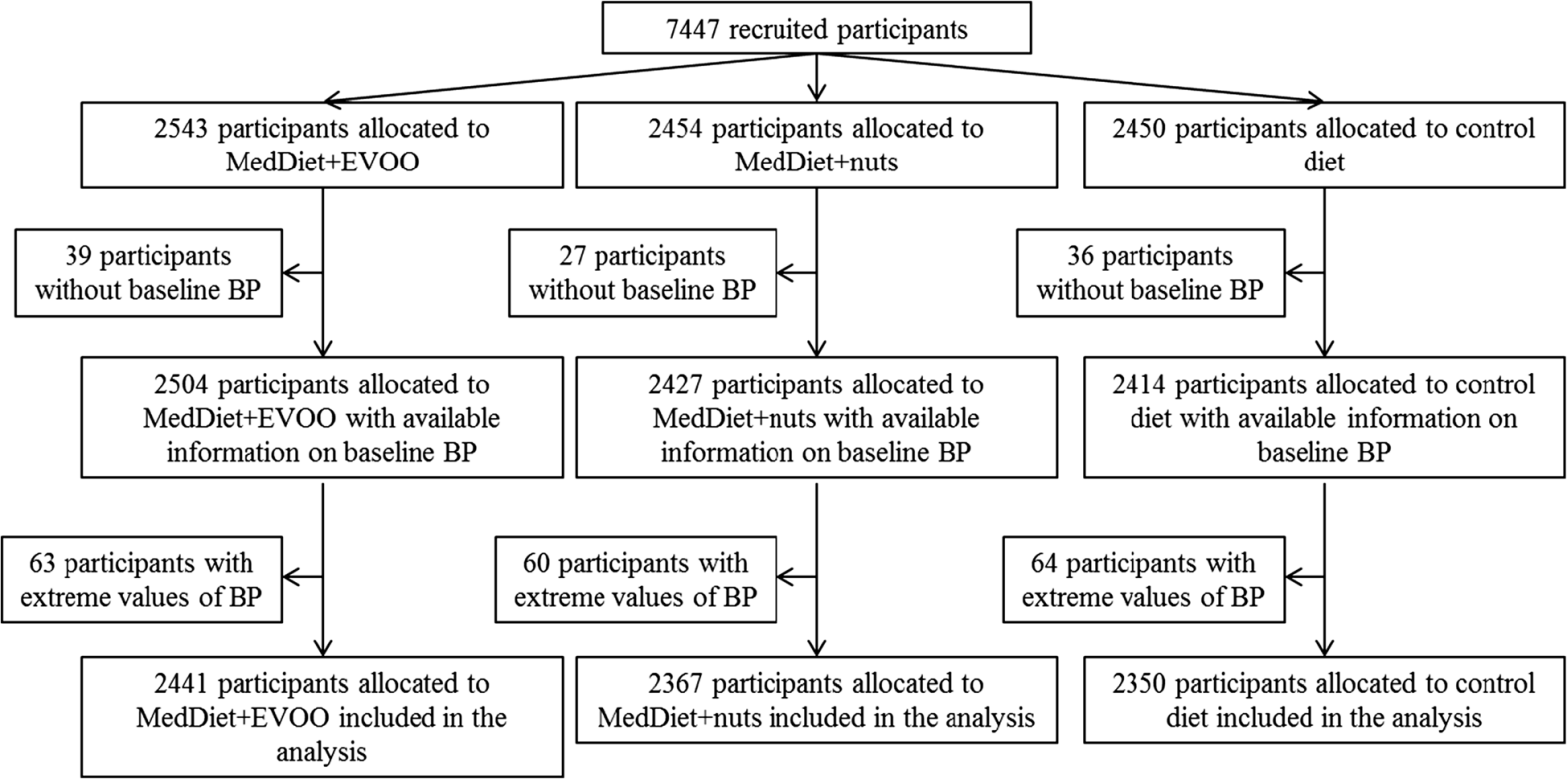
Flowchart of participants in the PREDIMED trial.

Of the 7,447 participants recruited to the PREDIMED trial, 289 were excluded either because there was no information on their baseline BP or they had extreme BP values. Thus, our effective sample size was 7,158 (Figure 1). On average, participants had 3.8 visits with available BP information during follow-up. Specifically, 2,345 participants in the MD+EVOO group, 2178 participants in the MD+nuts group, and 2,064 participants in the control group had BP measurements during follow-up.

In the PREDIMED trial, slightly more women than men were recruited. Mean age was 67 years. Participants were at high cardiovascular risk as per protocol, and they had an average baseline MD score of 8.6 in the 14-point score of adherence to the MD. All three groups were well balanced in their baseline characteristics, including their dietary and non-dietary traits (Table 1).

**Table 1:** Baseline characteristics of the PREDIMED trial participants according to intervention group

**Characteristic**	**MD+EVOO, n = 2441**	**MD+nuts, n = 2367**	**Control diet, n = 2350**
Female sex, n (%)	1424 (58.3)	1275 (53.9)	1402 (59.7)
Age, years	66.9 ± 6.2	66.6 ± 6.1	67.3 ± 6.3
Smoking habit, n (%)			
Never-smoker	1505 (61.7)	1414 (59.7)	1462 (62.2)
Former smoker	599 (24.5)	613 (25.9)	561 (23.9)
Current smoker	337 (13.8)	340 (14.4)	327 (13.9)
Body mass index^a^	29.9 ± 3.7	29.7 ± 3.8	30.2 ± 4.0
Waist circumference, cm	100 ± 10	100 ± 11	101 ± 11
Hypertension, n (%)^b^	1999 (81.9)	1951 (82.4)	1972 (83.9)
Systolic BP, mmHg	148 ± 19	149 ± 18	149 ± 19
Diastolic BP, mmHg	83 ± 10	83 ± 10	82 ± 10
Anti-hypertensive therapy, n (%)	1660 (68.0)	1648 (68.4)	1666 (70.9)
Type 2 diabetes, n (%)^c^	1224 (50.1)	1092 (46.1)	1127 (48.0)
Treatment for type 2 diabetes, n (%)	801 (32.8)	715 (30.2)	786 (33.5)
Dyslipidemia, n (%)^d^	1755 (71.9)	1741 (73.6)	1697 (72.2)
Lipid-lowering therapy, n (%)	1095 (44.9)	1041 (44.0)	1032 (43.9)
Family history of premature CHD, n (%)^e^	553 (22.7)	514 (21.7)	538 (22.9)
MD adherence score^f^	8.7 ± 2.0	8.8 ± 2.0	8.4 ± 2.0
Dietary sodium intake, g/day	2.4 ± 0.9	2.4 ± 0.9	2.3 ± 0.9
Dietary potassium intake, g/day	4.4 ± 1.2	4.4 ± 1.1	4.2 ± 1.1
Dietary calcium intake, g/day	1.1 ± 0.4	1.1 ± 0.4	1.0 ± 0.4

CHD, coronary heart disease; MD+EVOO, Mediterranean diet plus extra virgin olive oil; MD+nuts, Mediterranean diet plus nuts.

Values are mean ± SD unless otherwise specified.

^a^Body mass index is the weight in kilograms divided by the square of the height in meters.

^b^Hypertension was defined as systolic BP ≥140 mm Hg, diastolic BP ≥90 mm Hg, or use of anti-hypertensive therapy.

^c^Diabetes was defined as fasting blood glucose ≥126 mg/dl (7.0 mmol/l) on two occasions, or 2-hour plasma glucose ≥ 200 mg/dl (11.1 mmol/l) after a 75 g oral glucose load, or use of anti-diabetic medication.

^d^Dyslipidemia was defined as low-density lipoprotein cholesterol >160 mg/dl, high-density lipoprotein cholesterol ≤40 mg/dl in men or ≤50 mg/dl in women, or use of lipid-lowering therapy.

^e^A family history of premature CHD was defined as diagnosis of the disease in a male first-degree relative before the age of 55 years or in a female first-degree relative before the age of 65 years.

^f^MedDiet adherence score (minimum adherence = 0 points; maximum adherence = 14 points).

**Figure 2:**
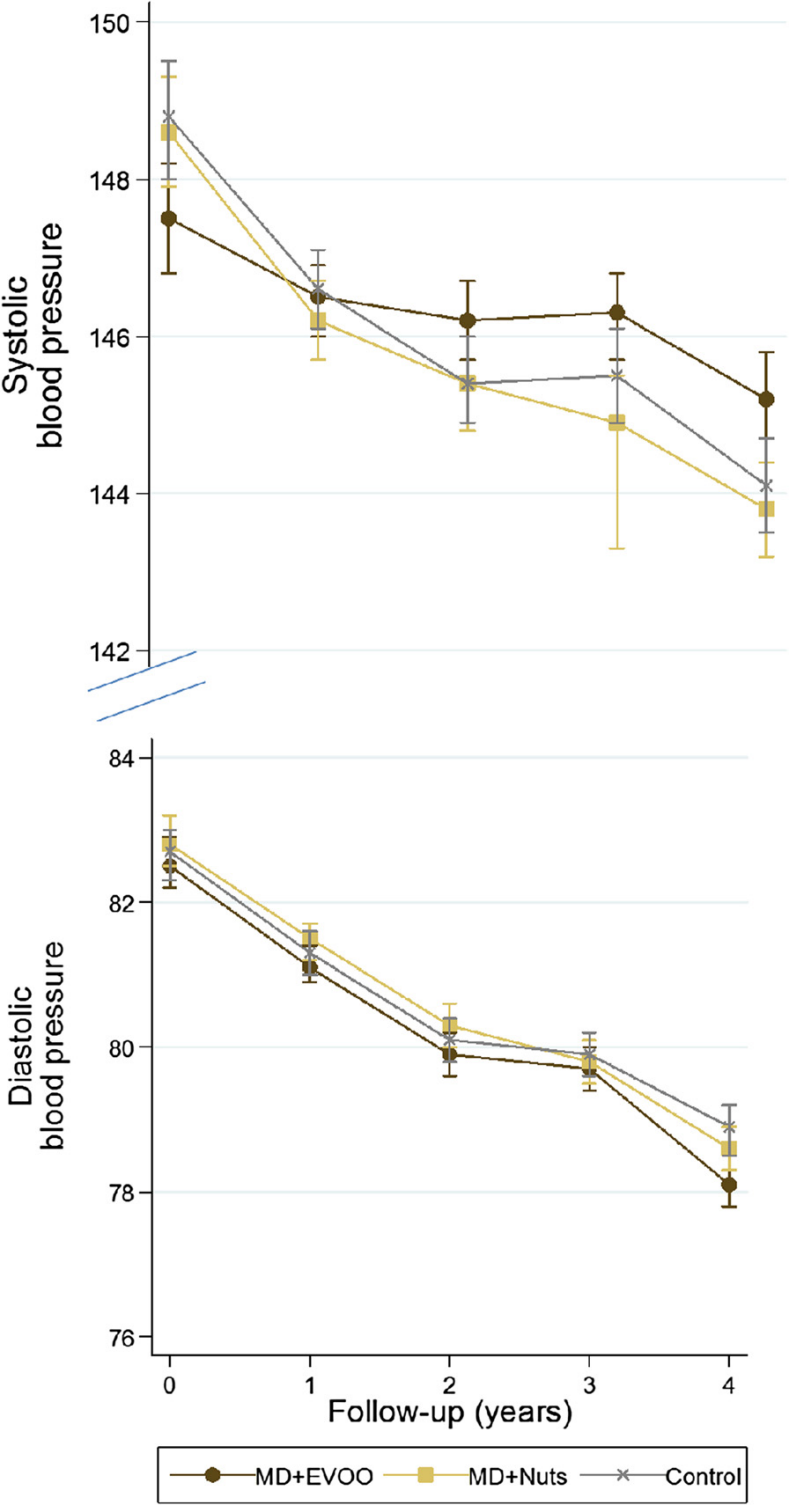
**Adjusted mean systolic and diastolic blood pressure at baseline and yearly visits according to intervention group. **Values are adjusted for center, sex, age, type 2 diabetes and baseline blood pressure.

Significant reductions in both systolic and diastolic BP were apparent for the three randomized groups during follow-up (*P*<0.001 for within-group changes during follow-up time, adjusted for center, sex, age, and baseline T2D) (Figure 2). There were no significant between-group for systolic (*P* = 0.51 for MD+EVOO versus control and *P*>0.99 for MD+nuts versus control) or diastolic(*P* = 0.39 for MD+EVOO versus control and *P* = 0.09 for MD+nuts versus control) BP. These latter comparisons were adjusted for center, sex, age, and baseline T2D.

We found a greater reduction in average systolic BP in the MD+nuts group than in the control group. However, between-group differences in systolic BP versus control with up to 4 years of follow-up were apparent only in crude analyses, and they became non-significant after multivariate adjustment. No differences in systolic BP were found between the MD+EVOO and the control group (Table 2). However, compared with the control group, greater reductions in diastolic BP were seen for both MDs. These differences remained significant in multivariate-adjusted analyses, with adjusted differences of −1.53 mmHg (95% CI −2.01 to −1.04) for MD+EVOO versus control and −0.65 (95% CI −1.15 to −0.15) mmHg for MD+nuts versus control.

**Table 2:** Mean differences in BP changes (mmHg) in the two intervention groups versus the control group after 4 years of follow-up (median follow-up 3.8 years)

	**MD+EVOO versus control**	***P *value**	**MD+nuts versus control**	***P *value**
Systolic BP				
Crude model	0.42 (−0.46 to 1.30)	0.35	−0.90 (−1.77 to −0.03)	0.04
Multivariate-adjusted 1^a^	0.40 (−0.46 to 1.27)	0.36	−0.73 (−1.58 to 0.13)	0.10
Multivariate-adjusted 2^b^	0.41 (−0.46 to 1.28)	0.35	−0.72 (−1.57 to 0.13)	0.10
Multivariate-adjusted 3^c^	0.39 (−0.48 to 1.26)	0.38	−0.72 (−1.58 to 0.13)	0.10
Diastolic BP				
Crude model	−1.41 (−1.92 to −0.91)	<0.001	−0.61 (−1.12 to −0.09)	0.02
Multivariate-adjusted 1^a^	−1.49 (−1.98 to −1.00)	<0.001	−0.65 (−1.15 to −0.14)	0.01
Multivariate-adjusted 2^b^	−1.49 (−1.97 to −1.00)	<0.001	−0.64 (−1.15 to −0.14)	0.01
Multivariate-adjusted 4^d^	−1.53 (−2.01 to −1.04)	<0.001	−0.65 (−1.15 to −0.15)	0.01

BP, blood pressure; MD+EVOO, Mediterranean diet plus extra virgin olive oil; MD+nuts, Mediterranean diet plus nuts.

^a^Adjusted for center, age, sex and diabetes.

^b^Multivariate-adjusted 1 + additional adjustment for number of baseline anti-hypertensive drugs.

^c^Multivariate-adjusted 2 + additional adjustment for baseline systolic BP.

^d^Multivariate-adjusted 2 + additional adjustment for baseline diastolic BP.

Improvements in BP control were apparent for all three groups. The percentage of participants who attained appropriate control of BP levels significantly increased during follow-up time in all the three intervention groups (*P*<0.001 for time) (Table 3). This beneficial significant within-group change was maintained after adjusting for center, age, sex, and baseline T2D. No significant between-group differences were seen.

**Table 3:** **Percentage of participants with controlled BP levels (systolic BP <140 mmHg and diastolic BP <90 mmHg) during follow-up in the PREDIMED trial**^a^

	**Intervention group**						
	**MedDiet+EVOO**	***P *value**^**b**^	**MedDiet+nuts**	***P *value**^**b**^	**Control**	***P *value**^**b**^	***P *value**^**c**^
Baseline	33.6 (31.7 to 35.5)	–	31.1 (29.3 to 33.0)	–	31.1 (29.2 to 33.0)	–	NA
Year 1	36.2 (34.2 to 38.2)	–	36.9 (34.8 to 39.0)	–	37.2 (34.9 to 39.4)	–	>0.99
Year 2	38.6 (36.5 to 40.7)	–	40.4 (38.2 to 42.6)	–	41.4 (38.9 to 43.9)	–	0.35
Year 3	37.8 (35.7 to 40.0)	–	39.2 (36.8 to 41.5)	–	39.0 (36.4 to 41.5)	–	>0.99
Year 4	39.9 (37.4 to 42.3)	<0.001	41.5 (38.8 to 44.3)	<0.001	42.6 (39.5 to 45.7)	<0.001	0.69

BP, blood pressure; MD+EVOO, Mediterranean diet plus extra virgin olive oil; MD+nuts, Mediterranean diet plus nuts; NA, not applicable.

^a^Adjusted for center, age, sex and diabetes.

^b^*P*-value for within-group changes.

^c^*P*-value for between-group changes, after adjustment for multiple comparisons with the Bonferroni method.

The average number of anti-hypertensive drugs prescribed for the PREDIMED participants significantly increased during follow-up in the three intervention groups after adjustment for center, sex, age and baseline T2D. We found no significant between-group differences. At the end of follow-up, the average number of BP-lowering drugs in the PREDIMED participants was 1.41 (95% CI 1.36 to 1.46) in the MD+EVOO group, 1.39 (95% CI 1.33 to 1.44) in the MD+nuts group, and 1.39 (95% CI 1.33 to 1.45) in the control group (*P*>0.99).

## Discussion

In this large randomized controlled trial, participants in all three groups showed improvement in their BP levels, and, consequently, the percentage of participants with controlled BP also increased in all three groups. However, a greater reduction in diastolic BP was obtained with the MD interventions than with control intervention (advice to follow a low-fat diet). This could partly explain the recently reported benefit of the MD intervention on clinical disease end-points [8], especially the reduction in incidence of stroke, a cardiovascular event clearly related to high BP. However, other mechanisms apart from BP also need to be taken into account [19].

BP tends to increase with age. Thus, had our participants not experienced any intervention, they would be expected to show an increase in their BP levels during the follow-up period [20]; however, they actually had a decrease in their BP levels during the intervention. It could be argued that participants in clinical trials undergo more evaluations, and thus their doctors are more likely to prescribe a better adjustment of their anti-hypertensive medication. However, it should be noted that the intervention was based only on dietary changes, and no adjustments in the participants’ regular prescriptions were part of the intervention. In addition, participants in the PREDIMED trial were recruited from their primary healthcare centers, therefore, they were already attending medical consultations regularly prior to study entry. In fact, the number of outpatient contacts in 2004 in Spain was higher than the average in the European Union [21]. In addition, our participants were already under medical treatment for their cardiovascular risk factors.

Current guidelines for the prevention and treatment of high BP recommend adhering to the DASH diet [22], which is a healthy eating plan low in saturated fat, cholesterol, and in total fat. This diet emphasizes the consumption of fruits, vegetables, and fat-free or low-fat milk and dairy products [5]. The basic recommendations provided to our participants in the control group had many aspects in common with the DASH eating plan, and was not similar to a conventional placebo. Therefore, it was foreseeable that participants in the control group would also improve their BP levels if they followed this advice. In fact, a cohort study with healthy young participants also conducted in Spain previously reported an inverse association between adherence to the DASH diet and incident hypertension [23]. Thus, had we had a ‘true’ control group (for example, with a typical Western dietary pattern, or with no intervention at all) the between-group differences both in stroke and BP would have been greater. Regarding the intervention groups, the traditional MD is also rich in fruits and vegetables, has low content of saturated fat and dietary cholesterol, and is rich in magnesium and potassium [24], thus, in spite of its high total fat content, the MD could enhance BP control. Even though greater adherence to the MD has shown no association with incident hypertension in some large cohorts [25], a meta-analysis of trials with the MD on the components of metabolic syndrome found beneficial effects on average systolic and diastolic BP levels [9]. Similarly, we found a significant decrease in systolic and diastolic BP in both MD groups. Even though the intervention did not target sodium intake, participants in the PREDIMED trial on the whole did experience a significant reduction in their average sodium intake, as measured by the semi-quantitative food-frequency questionnaire. In addition, we found between-group significant differences (*P*<0.001) in sodium reductions favoring the two MD groups. Specifically, participants in both intervention (MD) groups experienced greater sodium reductions than did participants in the control group. However, these differences are unlikely to explain the observed results, as 1-year changes in sodium intake were not significantly associated with 1-year changes in BP after adjustment for major confounders, including the allocation group (data not shown). In addition, changes in potassium or calcium were also significantly associated with changes in BP in the multivariate analyses (data not shown). When we compared the two intervention groups with the control group, we found a significantly larger decrease in diastolic BP in both MD groups than in the control (low-fat) group. These results suggest that the MD may have a greater effect on diastolic BP control than a low-fat diet. Even though the between-group differences may seem small, it has been estimated that small differences in BP may have a large influence on cardiovascular and total mortality [26]. This influence needs to be considered within the context of the population strategy for preventive medicine [27]. Considering the strong association between diastolic BP and vascular mortality [28], these results have important clinical relevance but need to be taken in consideration when explaining the mechanisms of CVD risk reduction of the MD.

Our results may not seem in perfect agreement with our previously published results in a small subsample of PREDIMED participants (our pilot study) after only a 3-month follow-up. Greater reductions in systolic and diastolic BP were then seen in both MD groups compared with the control group [29]. There may be several explanations for these differences. First, only the first participants recruited for the trial were included in the pilot study. Second, the current work is based on a longer follow-up than the pilot study, therefore, a different and longer induction period is assumed. Third, in 2006 the protocol was reviewed; prior to 2006, no active nutritional education was given to the control group to foster their adherence to the low-fat diet, and they received only an information brochure. After the protocol review, an educational intervention was also devised and implemented for participants in the control group to promote the adherence to a low-fat diet with similar methodology to that of the two MD groups. Fourth, a higher rate of loss to follow-up occurred in the control group than in the two MD groups. It is possible that the participants retained in the control group had a healthier profile, as suggested by their baseline information [8]; this would selectively bias the results in the control group towards better BP levels had all participants in this group been followed up.

The present study has several limitations. First, changes in BP were a secondary end-point, not the primary end-point of the PREDIMED trial. Nevertheless, changes in BP were included in the protocol as a secondary specific aim from the very beginning of the trial design. Second, at baseline, a initial fair level of adherence to the MD was present in all participants, regardless of their allocated group, and participants in the control group also maintained their relatively high scores of adherence to the traditional MD during the study [8]. Therefore, the magnitude of attained between-group differences in adherence to the MD during follow-up was not large. These modest differences can be explained because for most participants their baseline diet was similar to the trial Mediterranean diet. In addition, even though participants in the control group received advice to reduce fat intake, changes in total fat were small, and the largest differences at the end of the trial were in the distribution of fat subtypes. The good quality of the diet in the control group may have impaired our ability to find large between-group differences in BP changes. Notwithstanding, a significantly better adherence to the prescribed diet was found in the two MD groups than in the control group, and after the first follow-up year, mean scores of adherence to the prescribed diet were significantly higher in the two MD groups than in the control diet group (*P*<0.001 for all yearly comparisons from years 1 to 4 of follow-up). After 3 years of follow-up we found significantly better scores in both MD groups than in the control group for 12 of the 14 items included in the MD adherence screening questionnaire. Therefore, a modest change in many aspects of the overall dietary pattern, and not only in the provided supplemental foods, was achieved with our intervention. Third, information on BP during follow-up was not available for a subset of participants, especially in the control group. As has been already published, participants for whom this information was not available during follow-up had a worse cardiovascular profile at study inception than participants who were retained, suggesting a bias toward a benefit in the control group [8]. Fourth, our participants lived in a Mediterranean country, had a high cardiovascular risk, and were mainly hypertensive subjects; all these characteristics may limit the generalizability of our findings. Fifth, information on anti-hypertensive drugs dosage was not available, and this precluded a detailed analysis on anti-hypertensive drug usage. However, because all participants usually attend consultations with their primary healthcare providers, it is unlikely that participants in one or the other group would be differentially treated.

The strengths of the study include the randomized design, the long duration of the intervention, the high compliance of the participants allocated to the MD with the intended intervention, the large study size, and the uniformity of study implementation across the different study sites.

## Conclusions

In conclusion, our randomized trial conducted in patients at high risk of CVD supports the traditional MD supplemented with either EVOO or nuts and the control diet as having beneficial effects on BP. After 4 years of follow-up, lower values of diastolic BP were seen in the two groups that received an intervention with a traditional MD supplemented with either EVOO or with nuts than in the control group.

## Abbreviations

BP: Blood pressure; CHD: Coronary heart disease; CI: Confidence interval; CVD: Cardiovascular disease; MD: Mediterranean diet; PREDIMED: Prevencion con Dieta Mediterranea.

## Competing interests

RE has served on the board of and received lecture fees from the Research Foundation on Wine and Nutrition (FIVIN); served on the boards of the Beer and Health Foundation and the European Foundation for Alcohol Research (ERAB); received lecture fees from Cerveceros de España and Sanofi-Aventis; and received grant support through his institution from Novartis. ER has served on the board of and received personal travel support as well as grant support through his institution, from the California Walnut Commission; served on the board of the Flora Foundation (Unilever); served on the board of and received lecture fees from Roche; served on the board of and received grant support through his institution from Amgen; received consulting fees from Damm and Abbott; received consulting fees and lecture fees, as well as grant support through his institution, from Merck; received lectures fees from Danone, Pace, AstraZeneca, and Rottapharm; received lecture fees and payment for the development of educational presentations, as well as grant support through his institution, from Ferrer; received payment for the development of educational presentations from Ricordati; and received grant support through his institution from Sanofi-Aventis, Takeda, Daiichi Sankyo, Nutrexpa, Feiraco, Unilever, and Karo Bio. JS-S has served on the board of and received grant support through his institution from the International Nut and Dried Fruit Council; received consulting fees from Danone; and received grant support through his institution from Eroski and Nestlé. FA has received payment for the development of educational presentations from Menarini and AstraZeneca. RM L-R has served on the board of and received lecture fees from FIVIN; received lecture fees from Cerveceros de España; and received lecture fees and travel support from PepsiCo. LS-M has served on the boards of the Mediterranean Diet Foundation and the Beer and Health Foundation. SP has served on the board of and received grant support through his institution from the Residual Risk Reduction Initiative (R3i) Foundation; served on the board of Omega Fort; served on the board of and received payment for the development of educational presentations, as well as grant support through his institution, from Ferrer; received consulting fees from Abbott; received lecture fees, as well as grant support through his institution, from Merck and Roche; received lecture fees from Danone and Esteve; received payment for the development of educational presentations from Menarini; and received grant support through his institution from Sanofi-Aventis, Kowa, Unilever, Boehringer Ingelheim, and Karo Bio. No other potential conflict of interest relevant to this article is reported.

## Authors’ contributions

ET analysed and interpreted the data and was primarily responsible for writing the manuscript. FBH made substantial contributions to the study design and interpretation of data and critically reviewed the intellectual content. RE, PB-C, JS-S, and LS-M made substantial contributions to the acquisition of data and their interpretation, and to the trial implementation. DC, MIC, FA, EG-G, MF, JL, XP, and JVS substantially contributed to the acquisition of data and trial implementation. RML-R, GS, and VR-G substantially contributed to the study conception. MB revised the manuscript for important intellectual content. ER made substantial contributions to study design and interpretation of data. MAM-G substantially contributed to the study conception and design, trial implementation, amd acquisition and interpretation of data, and critically revised the manuscript for important intellectual content. All authors read and approved the final manuscript.

## Pre-publication history

The pre-publication history for this paper can be accessed here:

http://www.biomedcentral.com/1741-7015/11/207/prepub

## Supplementary Material

Additional file 1 Institutional Review Boards that approved the PREDIMED trial protocol. Click here for file
