# Supplementary material for: Effect of the Mediterranean diet on blood pressure in the PREDIMED trial: results from a randomized controlled trial
Source: BMC Med. 2013 Sep 19;11:207. doi: 10.1186/1741-7015-11-207 (PMC3849640; doi:10.1186/1741-7015-11-207)
Supplement: Additional file 1 — Institutional Review Boards that approved the PREDIMED trial protocol. [file 1741-7015-11-207-S1.docx]

The Research and Ethic Committee of the Hospital Clínic (Barcelona, Spain), accredited by the Department of Health and Human Services and regulated by the Federalwide Assurance for the Protection of Human Subjects of International (Non-US) Institutions # 00000738 approved the study protocol on 16 July 2002. In addition, the Institutional Review Boards of the other ten recruitment centers (see list below) reapproved the trial protocol:

- University of Navarra-School of Medicine
- University of Valencia
- University Rovira i Virgili
- Institut de Recerca Hospital del Mar
- University Hospital of Alava
- University of Malaga
- University of Balearic Islands, and Hospital Son Espases
- Department of Family Medicine, Primary Care Division of Sevilla
- University of Las Palmas de Gran Canaria
- Hospital Universitario de Bellvitge
